# Perceived stress and study-related behavior and experience patterns of medical students: a cross-sectional study

**DOI:** 10.1186/s12909-022-03182-4

**Published:** 2022-02-23

**Authors:** Kambiz Afshar, Birgitt Wiese, Stephanie Stiel, Nils Schneider, Bettina Engel

**Affiliations:** 1 Institute for General Practice and Palliative Care, Hannover Medical School, Carl-Neuberg-Straße 1, 30625 Hannover, Germany; 2 Division of General Practice/Family Medicine, Department for Health Services Research, School of Medicine and Health Sciences, Carl Von Ossietzky University Oldenburg, Ammerländer Heerstr. 114-118, 26129 Oldenburg, Germany

**Keywords:** Stress, Burnout, Medical education, Medical students, Cross-sectional studies

## Abstract

**Background:**

Distress and burnout are common in physicians. Both may already arise during medical training and persist throughout residency. An analysis of needs is necessary in order to develop target group specific curricular concepts at medical faculties. Aim of this study was to assess the perceived stress of medical students, to explore study-related behavior and experience patterns, and to investigate associated factors.

**Methods:**

We conducted a cross-sectional survey of medical students at the Hannover Medical School. The web-based questionnaire consisted of 74 items and included two standardized instruments: the “Work-related Behavior and Experience Patterns” (Arbeitsbezogene Verhaltens- und Erlebensmuster, AVEM) and the “Perceived Medical School Stress” scale (PMSS). Students were asked to state their self-perceived actual stress level on a scale from 0% (no stress at all) to 100% (maximum stress). We performed a classification and regression tree (CART) analysis to identify factors that can discriminate between the four different AVEM patterns.

**Results:**

Five hundred ninety-one medical students (female 75.8%, response rate: 34.0%) participated in the survey. The mean sum score of the PMSS was 37.2 (SD 8.3; median score 37, min.-max. = 18–65). Overall, 68.5% of the students showed a risk pattern (risk pattern A “overexertion”: 38.9%; risk pattern B “burnout”: 29.6%). Pattern G “healthy” was shown in 8.3% and pattern S “protection” in 23.1% of the students. Multilevel analysis revealed that the self-perceived stress level and the PMSS sum score were the most important predictors for the AVEM pattern assignment. Furthermore, academic year, gender, and financial dependency were relevant influencing factors: students in higher academic years with no financial support had a higher probability to be in risk pattern B whereas male students in the first academic year tended to be in pattern G.

**Conclusions:**

The PMSS sum score could objectify medical students’ high self-perceived stress level. The majority of participating students showed a risky study-related behavior and experience pattern. Medical faculties should be aware of the still existing and relevant problem of stress and burnout among medical students. Our results lay the groundwork for an evaluation and further development of medical curricula at the own faculty.

## Background

Distress and burnout are common in physicians [1]. Psychological strain of physicians may not only affect their own health but also the quality of their patient care [2]. To highlight the importance of physicians’ own health and well-being for high quality patient care, the Declaration of Geneva was revised in 2017 and amended by the following sentence: “*I will attend to my own health, well-being, and abilities in order to provide care of the highest standard*” [3].

Even though Freudenberger addressed occupational burnout for the first time half a century ago, many questions remain open [4]. Burnout is thought to be an occupational health problem involving multiple symptoms linked to chronic work-related stress, but has not been classified as a medical condition. In May 2019, the WHO clarified the definition of burnout, specifically connecting it to employment, rather than non-occupational life-management difficulties [5]. Maslach, Schaufeli and Leiter identified six risk factors for burnout: mismatch in workload, mismatch in control, lack of appropriate awards, loss of a sense of positive connection with others in the workplace, perceived lack of fairness, and conflict between values [6]. They postulated that burnout occurs when there is a disconnection between the organization and the individual with regard to these risk factors [6]. To resolve these discrepancies, integrated action is required on both levels the individual and the organizational.

An essential aspect is that stress and burnout already arise during medical training and often persist during residency; the prevalence of burnout varies from 7 to 70% [7–9]. Voltmer et al. showed that stress and the respective behavior patterns vary throughout medical education [10]. Negative factors contributing to the psychological strain of medical students are often related to changing academic stress, exams, and high workload [7]. Further studies have postulated that psychological stress is not only positively correlated with burnout but also affects the academic and professional performance of medical students and physicians [11, 12]. A recent study demonstrated the correlation between burnout, distress, and neuroticism as a personality trait: the level of burnout increased directly with the rise of psychological distress and academic stress and indirectly with the level of neuroticism [13]. In contrast, emotional intelligence seems to have protective effects on burnout, but is also reduced by psychological distress and neuroticism [13]. Different studies have also shown that there is a relevant prevalence of depression or depressive symptoms as well as suicidal ideation among medical students [14], which are significantly correlated with perceived stress [15]. Therefore, it is reasonable to focus on medical students and to promote health and wellbeing as early as possible during their studies. Nevertheless, only few medical faculties in Germany have implemented the topic of medical students’ health and well-being in their curricula. Given the serious nature of potential consequences and the high prevalence among medical students, addressing psychological strain at an early stage is imperative to preserve mental health of future physicians. In order to develop target group specific interventions and curricular concepts at medical faculties, obtaining knowledge about how medical students perceive stress at the own medical faculty and understanding how they experience and deal with study-related stress is a crucial prerequisite.

Thus, the aim of the present study was to assess the perceived stress of German medical students and to investigate study-related behavior and experience patterns to cope with stress. Furthermore, the study aimed to investigate different associated influencing factors that predict the assignment of medical students in these patterns.

## Methods

### Study design

We performed an exploratory cross-sectional survey study at the Hannover Medical School (MHH), Germany. The survey was administered between December 2018 and January 2019. The *Strengthening the Reporting of Observational Studies in Epidemiology* (STROBE) statement [16] for cross-sectional studies was used to ensure comprehensive reporting.

### Setting

The model medical educational programme at the MHH was established in 2005 and focuses on an integrated, work- and patient-orientated medical education. In the academic year 2020/21, in total 2,273 medical students (female: 64.3%) were registered at the MHH [17]. The gender distribution is similar to the distribution at other medical faculties in Germany [18].

### Study population

All registered medical students at MHH from the academic year 1 to 6 were invited for participation per email by the office of the Dean of Studies. In 2018, each academic year comprised about 290 medical students. There were no exclusion criteria.

At the end of the survey, participants had the opportunity to take part in a raffle to win one of in total 100 book vouchers amounted to 20 euros. Offering a reward ought to increase the response rate. Information on the sociodemographic characteristics (age and gender) of non-responders was provided by the university administration.

### Data collection and instruments

The survey was administered and enrolled using SoSci Survey, a professional tool for online surveys. The web-based questionnaire consisted of 74 items and included items of two standardized instruments: (1) the Work-related Behavior and Experience Patterns (Arbeitsbezogene Verhaltens- und Erlebensmuster, AVEM) and (2) the German version of the Perceived Medical School Stress scale (PMSS-D).

#### Work-related behavior and experience patterns (AVEM)

The short form of the AVEM comprises 44 items and the following 11 dimensions: subjective significance of work, career ambition, tendency to overexert, striving for perfection, emotional distancing, resignation tendencies, offensive coping with problems, balance and mental stability, satisfaction with work, satisfaction with life, and experience of social support. These dimensions can be assigned to different types of work-related experience and behavior patterns: pattern G and S as well as the risk patterns A and B. The main pattern of a survey participant is determined by estimating the concurrence of the individual data score and the four reference profiles (weighted linear combination based on an algorithm of discriminant analysis). The four patterns can be described as follows [19]:

- Pattern G: “healthy”. The healthy pattern G is characterized by a good balance between the domains of resistance towards stress, emotional well-being and professional commitment.
- Pattern S: “protection”. Looking at the 11 dimensions measured with AVEM, pattern S shows lower scores in the dimensions of professional commitment and high scores in emotional distancing from work. Either this could be due to a relaxed attitude towards work or the detachment from work could also be an early sign of demotivation and frustration that may later lead to burnout [20].

In contrast to these first two patterns, the following two patterns are defined as risk patterns. They have been repeatedly shown to be correlated to symptoms of illness and poor health [19].

- Risk pattern A: “overexertion”. For participants with this behavior pattern the importance of work is very high. Lower scores in the ability to cope with stress and emotional wellbeing show the “costs” of this exhaustive behavior.
- Risk pattern B: “burnout”. This risk pattern comprises the core symptoms of burnout. Participants with this behavior pattern show low scores in the dimensions of professional commitment and in the dimensions related to the domain of resistance towards stress. In addition, scores for satisfaction with work and life as well as for social support are low.

#### Perceived medical school stress (PMSS-D)

The PMSS-D is the German version of a widespread stress questionnaire designed for use especially in medical schools. It addresses a wide range of stressors, including workload, competition, social isolation, and financial worries [11, 21]. The PMSS-D consists of 13 items. Each item is rated on a 5-point Likert-type scale (1 = „I strongly disagree “; 5 = „I strongly agree “). The higher the sum score of all items (range 13–65) the greater the likelihood for pathological perceived stress.

#### Further items

Additionally, we added questions on sociodemographic data, on substance use and time spend on learning and working out of regular study time at MHH (8.00–16.00 o’clock). Students were also asked to assess their self-perceived actual stress level on a scale from 0% (no stress at all) to 100% (maximum stress).

In a pretest with five team members of the institute, the total time required to complete the questionnaires was about 15–20 min.

### Data analysis

Sociodemographic data and the items of the PMSS-D were analysed descriptively using the software IBM Statistical Package for Social Sciences version 26 (SPSS Inc., Chicago, IL/USA). Descriptive statistics of quantitative data included the calculation of median and interquartile range (IQR), mean and standard deviation (SD), frequencies and percentages.

A definite assignment to a pattern of the AVEM was made if > 95% of the dimensions could be assigned to one of the four patterns. Otherwise, a tendential pattern assignment took place.

A classification and regression tree (CART) analysis was performed to identify factors that can discriminate between different AVEM patterns. We investigated the following possible influencing factors: gender, marital status, children present, other vocational training/study, academic year, substance use, stress level, PMSS-D sum score, as well as financial support. The CART method is based on recursive partitioning analysis; the aim is to form prediction rules by constructing binary trees. Splitting rules are used as criteria to select the best split at each node; in this analysis, we used the Gini index of diversity as a measure of node impurity as a splitting rule. A tenfold cross-validation was used to assess its quality of fit, accurately.

Missing items were replaced by mean value imputation if less than 10% of items were missing.

## Results

### Sociodemographic data

In total, 591 medical students (female 75.8%) at MHH took part in the survey (response rate approximately 34.0%). 36.8% of participants stated to live in a permanent partnership and 5.8% of them have children. Table 1 gives an overview of the sociodemographic characteristics of participating students. In total, 31 medical students (5.2%) stated to use stimulating substances, i.e. methylphenidate or cocaine. 20 students (3.4%) stated to use stimulating substances to meet study-related performance expectations.

**Table 1:** Sociodemographic data of medical students (*n* = 591)

Item	n	%
Gender		
Male	143	24.2
Female	448	75.8
Marital status		
Single	371	62.8
Partnership	174	29.4
Married	44	7.4
Divorced	2	0.3
Children present		
Yes	34	5.8
No	557	94.2
Academic year (*N* = 539)		
1	85	14.4
2	72	12.2
3	101	17.1
4	102	17.3
5	112	19.0
6	67	11.3
Previous completed studies		
Yes	145	24.5
No	446	75.5
Side job		
Yes	367	62.1
No	224	37.9
Financial support in accordance with the Federal Student's Assistance Act (BAFöG^a^)		
Yes	155	26.2
No	436	73.8
Financial support by other third parties, i.e. parents		
Yes	471	79.7
No	120	20.3
Substance use		
Yes	31	5.2
No	555	93.9
I can't / don't want to answer	5	0.8

^a^*BAFöG* Bundesausbildungsförderungsgesetz

Totals may differ from 100% due to rounding imprecision

### Perceived stress level

**Fig. 1:**
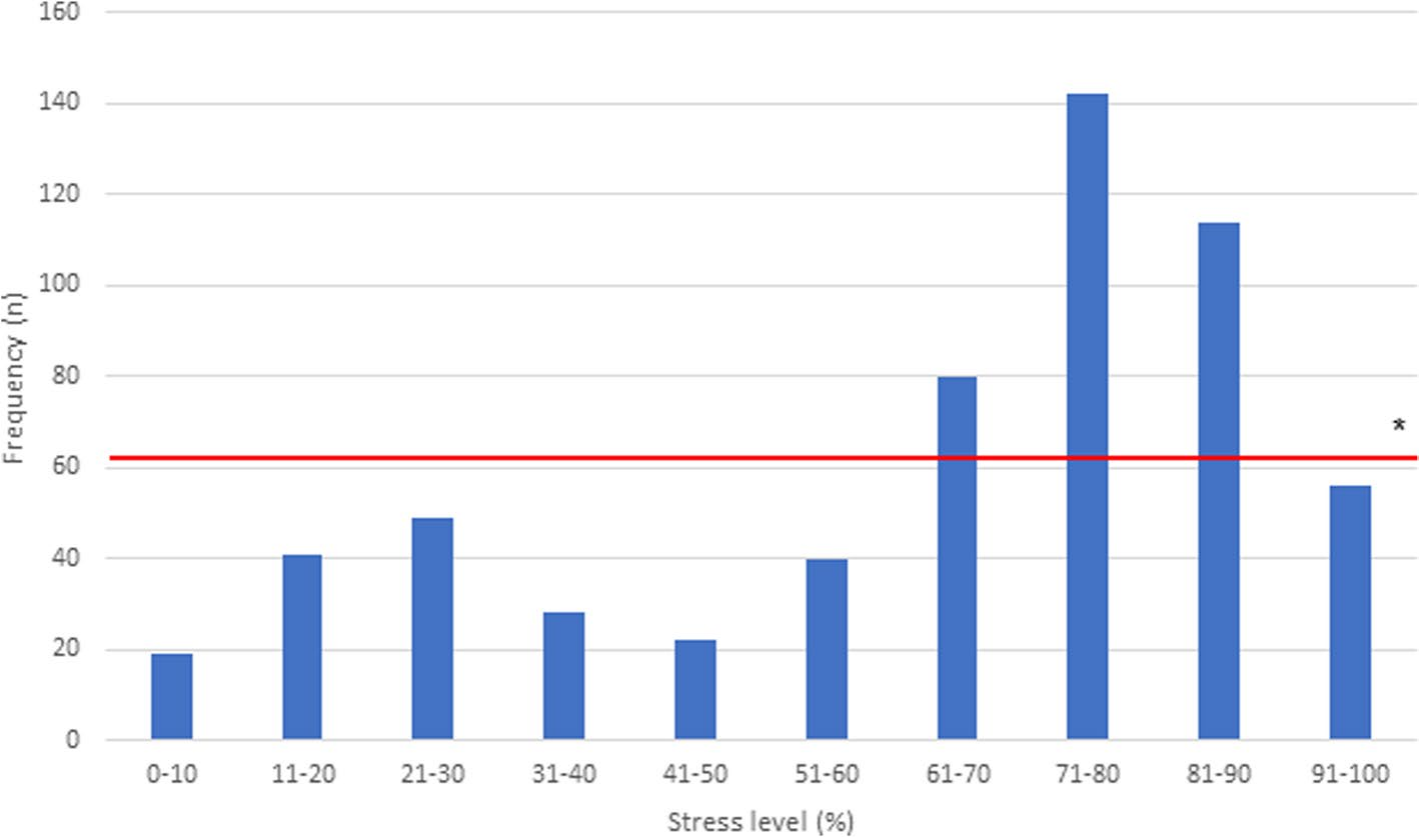
Medical students’ (*n*° = °591) subjective stress level (0–100%) * Mean stress level = 61.74%, standard deviation = 26.025

Students stated their currently experienced stress level in average with 61.7% (SD 26.0; median 71%, min.-max. = 1%-100%, see Fig. 1). Female students stated a higher average stress level than their fellow male students (70.5% vs. 53.1%). The mean sum score of the PMSS-D was 37.2 (SD 8.3; median score 37, min.-max. = 18–65).

### Study-related behavior and experience patterns

A definite assignment to a pattern of the AVEM was possible in 18.2% of the participating students. Overall, 68.5% of the students showed a definite risk pattern (pattern A: 38.9%; pattern B: 29.6%). Pattern G (“healthy”) was shown in 8.3% and pattern S (“protection”) in 23.1% of the students.

**Fig. 2:**
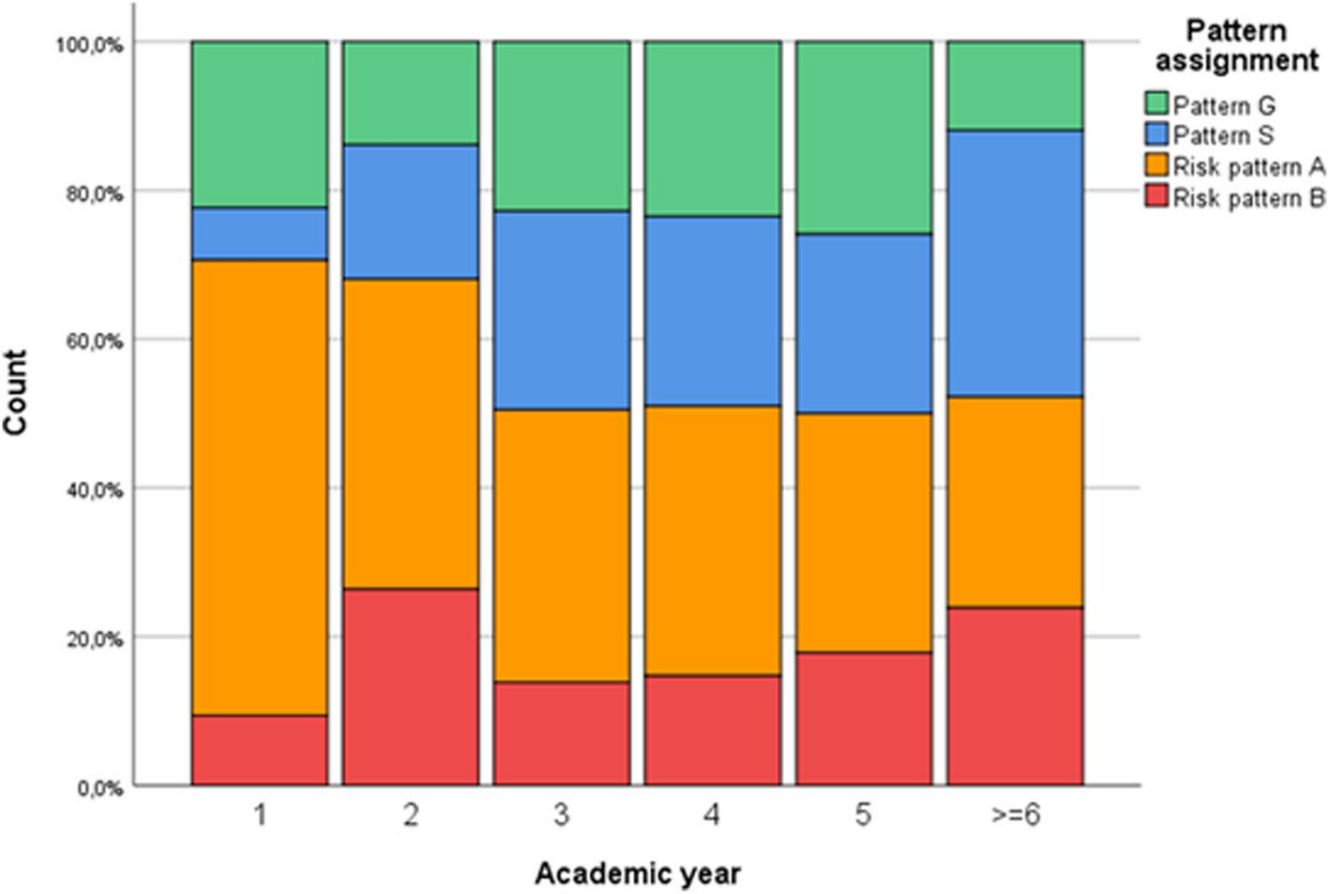
Medical students’ study-related behavior and experience pattern assignment (*n* = 591). Pattern G = “healthy”; Pattern S = “protection”; Risk pattern A = “overexertion”; Risk pattern B = “burnout”

With regard to the tendential pattern assignment, fewer students were in both risk groups (58.1% tendential risk pattern A (“overexertion”) and B (“burnout”), see Table 2). Comparing the pattern distribution in the different academic years, it is striking that both risk groups were significantly more pronounced in the first two academic years than in the following years (Fig. 2).

**Table 2:** Tendential assignment of study-related behavior and experience patterns in participating medical students (*n* = 591)

Pattern	n	%
Pattern G “healthy”	118	20.0
Pattern S “protection”	130	22.0
Risk pattern A “overexertion”	235	39.8
Risk pattern B “burnout”	108	18.3

Totals may differ from 100% due to rounding imprecision

### Multilevel analysis

**Fig. 3:**
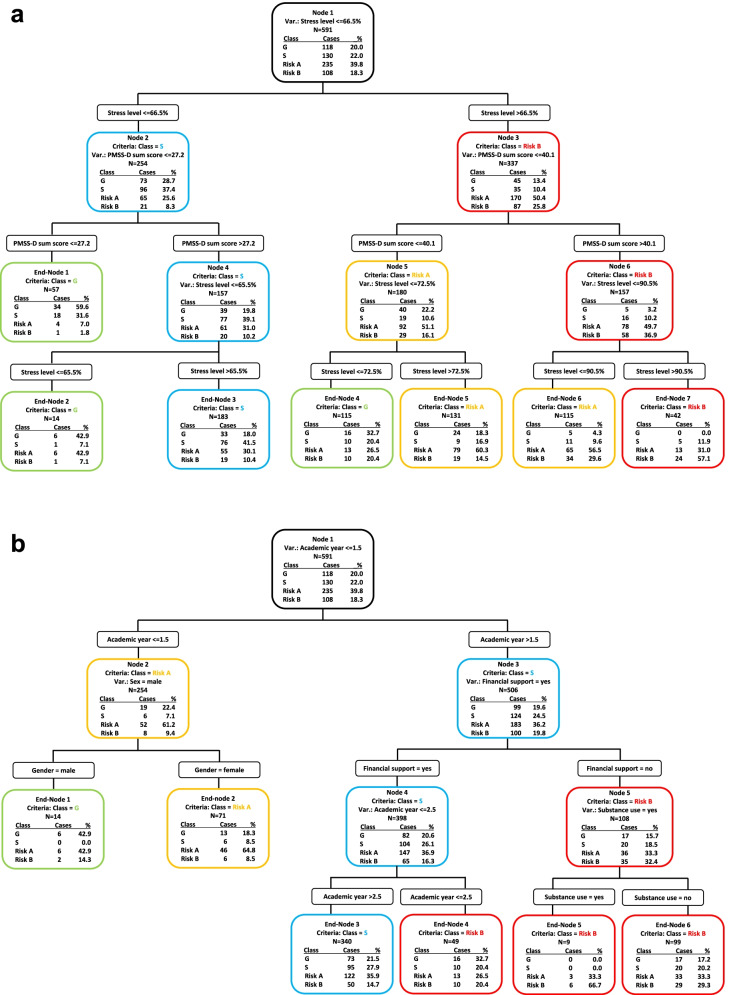
Classification and regression tree (CART) analysis. **a** Multivariate analysis with all parameters including stress level and PMSS-D sum score. **b** Multivariate analysis without stress level and PMSS-D sum score. G = pattern “healthy”; S = pattern “protection”; Risk A = risk pattern “overexertion”; Risk B = risk pattern “burnout”. Totals may differ from 100% due to rounding imprecision

The results of the CART analysis are shown in Fig. 3a and b.

Figure 3a shows the AVEM pattern distribution of all participating students (*n* = 591). The analysis shows a distribution made according to the self-perceived stress level and the PMSS-D sum score: higher values make an assignment to a higher risk group more likely. For example: Students with a self-perceived stress level of < = 66.5% and a PMSS-D sum score of < = 27.2 were more likely to be assigned to the AVEM pattern G "healthy" (59.6%; see “End-Node 1”). Whereas students with a self-perceived stress level of > 90.5% and a PMSS-D sum score of > 40.6 were more likely to be in risk group B (“burnout”) (57.1%; see “End-Node 7”). Figure 3a shows also a more detailed pattern distribution by further division, e.g. by self-perceived stress level margins of 66.5%, 72.5%, and 90.5%.

Figure 3b shows the distribution of the participating students in one of the four different AVEM patterns when the stress level and PMSS-D sum score were not taken into account. At first, students were divided by their academic year: Students in the first academic year were more often represented in risk pattern A (“overexertion”) (61.2%, see “Node 2”). These students were then divided by their gender: Female students were more likely to be represented in risk pattern A (“overexertion”) than their fellow male students (64.8%, see “End-Node 2” vs. 42.9%, see “End-Node 1”). Students in the second academic year or higher were distributed according to their financial support: Students without financial support were more likely to be in risk group B (“burnout”) (32.4%, see “Node 5”, vs. 16.3%, see “Node 4”). Students without financial support were then distributed according to substance use. Students who were at least in the second academic year, who did not receive financial support and stated substance use were all (*n* = 9) in risk group A (“overexertion”) (33.3%) or B (“burnout”) (66.7%), (see “End-Node 5”). All other parameters, i.e. marital status, children present, other vocational training/study showed no relevant influence in this model.

## Discussion

In this study, we assessed the perceived stress of German medical students and explored study-related behavior patterns to cope with stress. Furthermore, this study investigated different influencing factors that predict the assignment of medical students in these patterns.

Our results show that students stated a high subjective stress level that could be objectified by the PMSS-D sum score. The items of the PMSS-D are specific for the medical school context. Different international studies could show, that the PMSS is not only associated with anxiety and depression [22], it is furthermore a predictor of psychological strain after graduation [23].

The majority of participating students were in one the two risk patterns of the AVEM. The subjective stress level and the PMSS-D sum score were the most important predictors for the AVEM patterns. Furthermore, academic year, gender, and financial dependency were relevant influencing factors: students in higher academic years with no financial support have a higher probability to be in risk pattern B (“burnout”) whereas male students in the first academic year tended to be in pattern G (“healthy”).

The results indicate that there might be a change of study-related behavior and experience pattern from a risk pattern A (“overexertion”) that was predominantly shown in the first academic year to pattern S (“protection”) that was predominantly shown in the last academic year. Even though a longitudinal study design is necessary to prove this hypothesis and investigate the change of pattern distribution during the course of study, there are different explanatory approaches. Especially in the beginning of their studies, medical students are confronted with different life changing events, i.e. change of housing and life situation, moving to a different city, leaving family and friends, and financial concerns. Furthermore, they might experience a high workload, time pressure, existential situations and excessive demands expectations during their studies [24, 25]. Dealing with these challenges often result in decreased mindfulness to their own well-being and health, in increased psychological strain, and in a loss of empathy [26]. A small proportion of participating students stated substance abuse, partly in order to cope with study-related challenges and expectations. In comparison with data of medical students in the United States, the proportion of substance use in our study was similar [27].

Although medical faculties can play a key role in preserving the health of their medical students, only few medical faculties in Germany, e.g. the *University zu Lübeck* [28], have implemented curricular interventions to address this topic. Therefore, medical faculties should assume responsibility. Internationally, there are different recommendations for educational program designs to promote student well-being, e.g. a consensus statement of Australia and New Zealand [29].

According to our study results, students of almost all academic years seemed to be affected. Especially students in the first two academic years showed a risky behavior and experience pattern. Thus, the implementation of a longitudinal curriculum with an emphasis on the start of medical studies might be appropriate to promote the health and wellbeing, self-care, and resilience of all medical students at MHH. As “student health” is not a compulsory element of the medical licensing regulations in Germany, respective learning contents should be integrated in a cross-disciplinary teaching concept without additional workload and an evident benefit for medical students.

### Strengths and limitations

The proportion of students in each academic year is a strength of this study. The majority of study participants were female students (ratio female : male = 3 : 1). The gender distribution is comparable to the gender distribution in the student population at the MHH and other medical faculties in Germany [17, 18]. The CART analysis method applied in this study has several advantages over traditional methods, including logistic regression models. It is nonparametric; no assumptions are made regarding the underlying distribution of values of the discriminator with respect to predictor variables. It can handle numerical data that are highly skewed or multimodal, including categorical predictors. CART is often able to uncover complex interactions or patterns between predictors that may be difficult or impossible to uncover using traditional multivariate techniques.

The response rate of 34% is not very high, which could be explained partially by the highly sensitive topic itself. Experiencing academic stress and its related comorbid conditions such as burnout is still not popular in the high-performance setting of medical education. Furthermore, the lengths of the questionnaire including up to 77 items might have decreased the motivation of medical students to participate. In addition, we used only one reminder after one month as well as leaflets and posters to draw attention to the survey. Medical students receive many emails during the semester. It might be that several students did not read the invitation to participate or they prioritized very deliberately how to spend their valuable time. Nevertheless, an average response rate of approximately 30% is considered to be adequate for online-based survey studies [30, 31]. Due to potential selection bias, our results of this study cannot be generalized and applied to students at other medical faculties unreservedly. It is possible that only the particularly affected and stressed medical students participated in this study. The proportion of students with very low or very high stress level and of students with oral substance abuse might be underestimated due to social desirable response behavior.

### Prospect

As we conducted a cross-sectional study, further studies should investigate students’ individual development of psychological strain and study-related behavior pattern over the course of studies and during transition from medical training to medical residency. As a next step, multi-center studies are necessary to compare data of different medical faculties in order to differentiate which factors may effect student health on an individual and organizational level. To obtain a better understanding of potential causes for the development of distress and burnout throughout medical education and further training, a qualitative research design, e.g. with guided interviews or focus groups, would allow for an in-depth analysis.

## Conclusions

In our study, the PMSS sum score could objectify the high self-perceived stress level in German medical students at the MHH. The majority of participating students showed a risky study-related behavior and experience pattern. The results justify an evaluation of the existing medical curriculum at the own faculty in order to implement curricular offers to cope with stress and address the high percentage of medical students who are at risk of overexertion and  who may develop burnout symptoms.

Different strategies and intervention are recommended to cope with stress during medical education, e.g. student counselling, support of positive thinking, mindfulness training, or facilitated discussion groups [32, 33]. At the MHH, the topic of student health is not systematically implemented in the medical curriculum, yet. The results will form the groundwork for a target group specific implementation of curricular interventions and activities to promote health awareness and self-care of medical students. Aim of such interventions is that medical students feel safe to talk about psychological strain and mental health problems without feeling stigmatized or less intelligent [34]. Another aim of such interventions is the training of resilient future physicians who can take care of themselves and of their patients [35].

## Data Availability

The datasets used and/or analyzed during the current study are available from the corresponding author on reasonable request.

## Abbreviations

AVEM: Work-related Behavior and Experience Patterns
CART: Classification and regression tree analysis
PMSS-D: German version of the Perceived Medical School Stress scale
